# Effect of Sarcopenia on Pneumonia after Endoscopic Submucosal Resection in Patients Aged ≥65 Years: A Retrospective Study

**DOI:** 10.3390/cancers15194753

**Published:** 2023-09-27

**Authors:** Min-Yu Kim, So Yeon Kim, Hye Jung Shin, Ki Hong Kweon, Jooeun Park, Na Young Kim

**Affiliations:** 1 Department of Anesthesiology and Pain Medicine, Anesthesia and Pain Research Institute, Yonsei University College of Medicine, Seoul 03722, Republic of Korea; minyu92@yuhs.ac (M.-Y.K.); kimsy326@yuhs.ac (S.Y.K.); kihong1@yuhs.ac (K.H.K.); rlo2@yuhs.ac (J.P.); 2 Biostatistics Collaboration Unit, Department of Biomedical Systems Informatics, Yonsei University College of Medicine, Seoul 03722, Republic of Korea; hjshin105@yuhs.ac

**Keywords:** endoscopic submucosal dissection, pneumonia, sarcopenia, old age, sedation, hospital stay

## Abstract

**Simple Summary:**

Elderly patients have a higher risk of developing pneumonia due to a decrease in the swallowing function and the effectiveness of coughing. Sarcopenia is an age-related decrease in muscle mass and strength. We investigated the association between sarcopenia and incidence of pneumonia after endoscopic submucosal dissection (ESD) in patients aged ≥65 years. Patients with (*n* = 1571) and without sarcopenia (*n* = 1718) who underwent ESD for gastric neoplasm were included. Propensity score matching (PSM) was performed between the groups at a 1:1 ratio. The primary endpoint was the effect of sarcopenia on the incidence of pneumonia after ESD. Patients with sarcopenia had an increased risk of developing pneumonia after ESD, even after adjusting for other factors, resulting in a higher incidence of leukocytosis and a longer duration of post-ESD hospitalization. The combination of sarcopenia and age ≥73 years could be an effective predictive factor for screening high-risk groups for pneumonia after ESD.

**Abstract:**

We aimed to investigate the association between sarcopenia and incidence of pneumonia after endoscopic submucosal dissection (ESD) in patients aged ≥65 years. Patients with (*n* = 1571) and without sarcopenia (*n* = 1718) who underwent ESD for gastric neoplasm were included. Propensity score matching (PSM) was performed between the groups (*n* = 785) at a 1:1 ratio. The primary endpoint was the effect of sarcopenia on the incidence of pneumonia after ESD. Among the included patients, 2.2% (*n* = 71) developed pneumonia after ESD. After PSM, the incidence rate of pneumonia was significantly higher in patients with sarcopenia than that in patients without sarcopenia (*p* = 0.024). Sarcopenia and age ≥73 years were significantly associated with the incidence of pneumonia (sarcopenia and age <73 years, odd ratio (OR) = 1.22 [95% confidence interval (CI): 0.46–3.22]; sarcopenia and age ≥73 years, OR = 3.92 [95% CI: 1.79–8.74]). Patients with sarcopenia had an increased risk of developing pneumonia after ESD, even after adjusting for other factors, resulting in a higher incidence of leukocytosis and a longer duration of post-ESD hospitalization. The combination of sarcopenia and age ≥73 years could be an effective predictive factor for screening high-risk groups for pneumonia after ESD.

## 1. Introduction

Endoscopic submucosal dissection (ESD) is considered less invasive and more effective than gastric resection for the treatment of gastric neoplasms in elderly patients [1,2,3,4]. ESD enables en bloc complete resection of superficial gastrointestinal neoplasms, which is beneficial for accurate histopathologic examination [5,6,7]. However, ESD must be performed cautiously as it can result in adverse effects, such as perforation, bleeding, and pneumonia, leading to clinically worse outcomes that require additional management [8,9,10].

Pneumonia, an adverse effect that can develop following ESD, can be a fatal complication in old age. Approximately 2.2–11% of patients undergoing ESD develop post-procedural aspiration pneumonia [11,12]. Deep sedation, procedure time over 2 h, old age (>75 years), male sex, and the presence of cerebrovascular disease are some of the known risk factors for pneumonia after ESD [12]. In particular, elderly patients are at a higher risk of developing aspiration pneumonia due to longer operation time and decreased gag reflex [13]. Aspiration pneumonia is basically caused by impairments in the swallowing and cough reflex [14,15]. The strength and quality of the swallowing and respiratory muscles tends to decrease with aging [16,17]. This decline reduces the swallowing function and the effectiveness of coughing, which hinders the clearing of the airways. Muscle mass and strength decrease with aging; this phenomenon is known as sarcopenia [14,18].

Sarcopenia is the age-related progressive loss of skeletal muscle mass, in addition to the decrease in muscle strength, and also causes weakness in the respiratory muscles, such as the diaphragm and intercostal muscles [18,19]. Sarcopenia in the swallowing and respiratory muscles can be a risk factor for developing pneumonia in old age. In addition, sarcopenia is associated with physical disability, injury, and mortality and is an independent risk factor for postoperative complications in patients undergoing radical gastrectomy [20]. Some reports have described the effect of sarcopenia on the incidence of pneumonia after ESD in elderly patients. However, most of these reports involve small sample sizes or focus on very elderly individuals, and thus, the relationship has not been conclusively established [14,21,22,23]. Therefore, this retrospective study aimed to investigate the association between sarcopenia and the incidence of pneumonia after ESD in elderly patients.

## 2. Materials and Methods

### 2.1. Study Population and Design

This single-center, retrospective study included patients aged ≥65 years who underwent preoperative abdominal computed tomography (CT) and ESD for gastric neoplasm under anesthesiologist-directed sedation between September 2010 and May 2020. Patients with incomplete data and patients in whom ESD was discontinued due to severe fibrosis, advanced lesions, and ambiguous lesions were excluded from this study. Based on the presence of sarcopenia, patients were divided into two groups: Sarcopenia and No sarcopenia groups. Propensity score matching (PSM) was performed to match patients with sarcopenia (sarcopenia group) and without sarcopenia (No sarcopenia group) at a ratio of 1:1, using the following covariates: age, body mass index (BMI), sex, chronic obstructive pulmonary disease (COPD), old tuberculosis, procedure time, gross type of legion, and submucosal fibrosis.

### 2.2. Measurements and Definitions of Sarcopenia

The skeletal muscle mass was measured by a radiologist at the level of the third lumbar vertebra (L3) on a transverse, cross-sectional CT image obtained during preoperative staging workup [24]. Skeletal muscle mass was quantified using Hounsfield units (HU) with thresholds of −29 HU to 150 HU using a commercially available imaging software (Aquarius Intuition version 4.4.12, TeraRecon Inc., San Mateo, CA, USA) [25,26,27]. The measured areas (cm^2^) were normalized for height (m^2^) and were defined as the skeletal muscle index (SMI). [28,29] Sarcopenia at baseline was defined as an SMI ≤ 53.4 cm^2^/m^2^ for men and ≤38.5 cm^2^/m^2^ for women in accordance with the criteria used in the study by Prado et al. [26].

### 2.3. Data Collection

Data on the general characteristics of the patients (age, sex, BMI, SMI, underlying diseases, American Society of Anesthesiologists physical status, smoking history, and alcohol history), procedure characteristics (procedure and recovery time, gross type of lesion, location of the lesion, type of resection, tumor size, and submucosal fibrosis), post-ESD complications (fever, leukocytosis, antibiotics use, pneumonia, perforation, and duration of post-ESD hospitalization), and sedation by anesthesiologist were collected. Diagnosis of pneumonia were defined as those who showed the presence of new or progressive lung infiltrates or consolidation in chest radiography and received antibiotics treatment [30].

### 2.4. Statistical Analysis

Continuous variables are presented as the mean ± standard deviation, and categorical variables are presented as the counts and proportions of patients. Group comparisons were performed using Student’s *t*-test for continuous variables and Chi-squared test or Fisher’s exact test for categorical variables, as appropriate. Logistic regression was used to investigate the relationship between sarcopenia and three dependent variables: pneumonia, fever, and leukocytosis. As the data were collected retrospectively, PMS was performed to achieve a balanced distribution of patient characteristics between the two groups and to adjust for the effects of confounding factors. The propensity score was calculated by the logistic regression model, in which the group was regressed according to age, sex, BMI, COPD, old tuberculosis, procedure time, gross type of lesion, and submucosal fibrosis. The patients were matched at a 1:1 ratio using the caliper matching method within 0.20 of the standard deviation of the logit of the propensity score. Paired *t*-test, McNemar’s test, and conditional logistic regression were used for the matched data. The optimal cutoff value for age was determined using the Youden index method on receiver operating characteristics (ROC) curve. *p*-values were two-sided and *p* < 0.05 was considered statistically significant. Statistical procedures were performed using SAS version 9.4 (SAS Institute, Cary, NC, USA) and R package (v. 4.0.4, http://www.r-project.org/ accessed on 1 July 2022) software.

### 2.5. Ethical Statement

This single-center, retrospective study was conducted after obtaining approval from the Institutional Review Board and Hospital Research Ethics Committee of the Yonsei University Health System, Seoul, Republic of Korea (IRB protocol No. 4-2020-1395) for retrospective data collection. The requirement for informed consent was waived by the IRB owing to the retrospective nature of the anonymous data. This study was conducted in accordance with the current version of the Declaration of Helsinki.

## 3. Results

**Figure 1:**
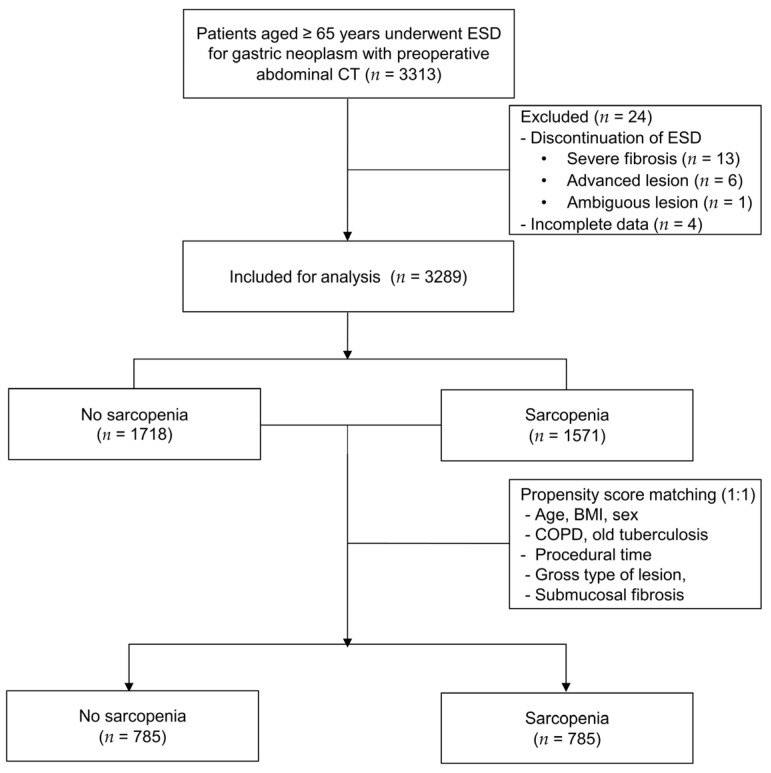
Flow diagram of patient selection. ESD, endoscopic submucosal dissection; BMI, body mass index; COPD, chronic obstructive pulmonary disease.

Among the 3313 patients identified initially, 13 patients with severe fibrosis, 6 patients with advanced lesions, 1 patient with ambiguous lesions, and 4 patients with incomplete data were excluded. Thus, 3289 patients were included in the analysis, among whom 1571 had sarcopenia. PSM was performed to match patients with and without sarcopenia at a 1:1 ratio (785 and 785, respectively) (Figure 1).

In the analysis of all patients, the incidence rate of pneumonia after ESD was 2.2% (71/3289 patients). The demographic and clinical characteristics of the patients were shown in Table 1. Compared to the patients without pneumonia, patients with pneumonia were more likely to have older age, male sex, and sarcopenia (*p* = 0.010, 0.003, and 0.015, respectively). The incidence of fever, leukocytosis, antibiotic use, and a longer duration of post-ESD hospitalization was more frequent in patients who developed pneumonia than that in those without pneumonia (*p* < 0.001, each). All patients were treated with medication, which was effective for all of them. Among the 3218 patients without pneumonia, 1527 patients had sarcopenia (48%). Among the 71 patients with pneumonia, 44 patients had sarcopenia (62%).

**Figure 2:**
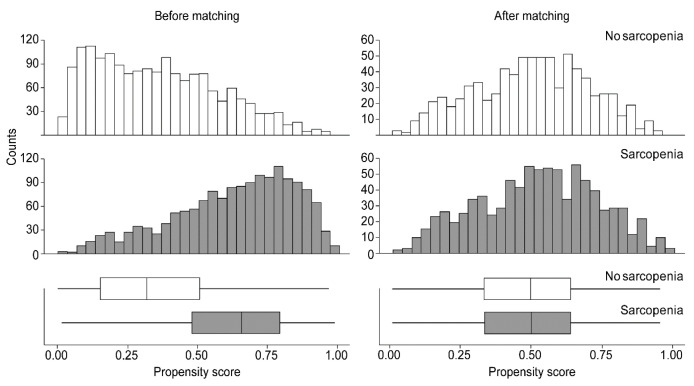
Distribution of the propensity scores of the patients with and without sarcopenia before and after matching.

The distributions of patients with and without sarcopenia were fairly uniform after PSM (Figure 2).

After matching, each group included 785 patients (Table 2). The SMI of the Sarcopenia and No sarcopenia groups were 44.7 ± 6.1 cm^2^/m^2^ and 54.0 ± 7.0 cm^2^/m^2^, respectively. No significant differences were observed between the baseline characteristics of the two groups, except for the number of patients with hypertension. The incidence rate of pneumonia was significantly higher in the Sarcopenia group than that in the No sarcopenia group (*p* = 0.024). In addition, the duration of post-ESD hospitalization and the incidence of leukocytosis and antibiotic use were higher in the Sarcopenia group than those in the No sarcopenia group (*p* = 0.048, 0.026, and 0.012, respectively).

**Figure 3:**
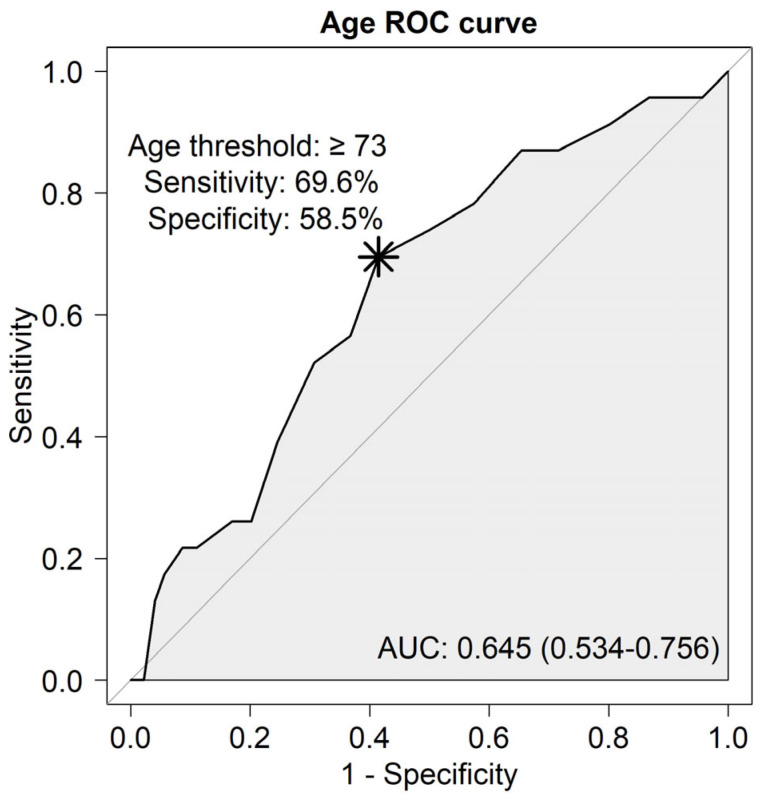
ROC curve analysis: the optimal cutoff value of age for pneumonia occurrence. ROC, receiver operating characteristics; AUC, areas under the receiver operating characteristics curves *.

The optimal cutoff value of the age was 73, which was determined using the Youden index method on the ROC curve (Figure 3).

When patients were classified according to sarcopenia and age, the probabilities of pneumonia were as follows: Sarcopenia and age <73 years, odd ratio (OR) = 1.22 [95% confidence interval (CI): 0.46–3.22]; sarcopenia and age ≥73 years, OR = 3.92 [95% CI: 1.79–8.74] (Table 3).

**Figure 4:**
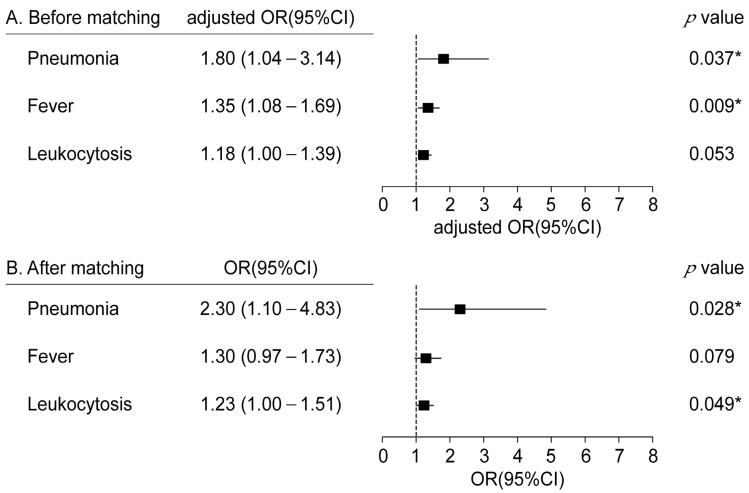
Forest plot of the odds ratio for pneumonia, fever, and leukocytosis (**A**) Before matching (**B**) after matching. * *p* < 0.05. CI, confidence interval; adjusted OR, adjusted odd ratio for age, body mass index (BMI), sex, chronic obstructive pulmonary disease (COPD), old tuberculosis, procedure time, gross type of legion, and submucosal fibrosis.

Logistic regression revealed that sarcopenia was a significant risk factor for the incidence of pneumonia and leukocytosis (Figure 4). The incidence of pneumonia and fever in the Sarcopenia group was significantly higher than that in the No sarcopenia group before PSM. After PSM, the sarcopenia group was associated with a significantly higher incidence of pneumonia (pneumonia, OR: 2.30, 95% CI: 1.10–4.83, *p* < 0.028) and leukocytosis (OR: 1.23, 95% CI: 1.00–1.51, *p* < 0.049).

## 4. Discussion

This study is the first case-matched retrospective study to evaluate the effect of sarcopenia on the incidence of pneumonia after ESD in patients over the age of 65 years. A significantly higher incidence of pneumonia after ESD was observed in the Sarcopenia group. Sarcopenia and age ≥73 years were predictive factors for the incidence of pneumonia after ESD. A higher risk of pneumonia in the Sarcopenia group resulted in a higher incidence of leukocytosis and antibiotic use and a longer duration of post-ESD hospitalization.

Aspiration pneumonia is one of the common post-procedure complications that can prolong hospitalization and increase mortality [31]. However, the risk factors for aspiration pneumonia after ESD have not been clearly identified [32]. According to the results of the present study, the incidence of pneumonia was significantly higher (2.9%) in patients with sarcopenia than that in those without sarcopenia (1.3%). Compared with the patients in the No sarcopenia group, patients in the Sarcopenia group had a 2.3-fold higher risk of developing pneumonia (*p* = 0.028). Previous studies have suggested old age, male sex, current smoking, pulmonary disease, cerebrovascular disease, remnant stomach, long procedural duration, and deep sedation as risk factors for aspiration pneumonia after ESD [12,32]. However, lesion or procedure-related risk factors are usually observed during the procedure, which makes the identification of high-risk patients before sedation challenging. In contrast, preoperative evaluation usually includes abdomen CT, which also provides lumbar muscle images. This makes sarcopenia more effective tool without extra imaging evaluation.

Sarcopenia is a systemic change occurring with aging that is associated with physical disability, injury, and mortality. With aging, changes in the regenerative capacity, protein turnover, fat and fibrotic composition of the muscles, reactive oxygen species, mitochondria function, and inflammation result in sarcopenia [33,34]. Although sarcopenia is an age-related issue, the cutoff age for sarcopenia-related complications is unknown. This study included patients aged ≥65 years, several previous studies have observed possible mechanisms of sarcopenia in patients aged ≥65 years. The postprandial amino acid availability, muscle perfusion, and digestive capacity are reduced in patients aged ≥65 years, thereby increasing the risk of anabolic resistance [35]. In men aged ≥65 years, higher testosterone levels were associated with reduced lean muscle loss and reduced visceral fat redistribution [36]. In the present study, patients with both sarcopenia and age ≥73 years had a 3.92-fold higher risk of developing pneumonia (*p* = 0.001). Therefore, patients aged ≥73 years with sarcopenia must be monitored carefully for pneumonia after ESD.

After matching, other than the SMI, hypertension differed significantly between the two groups (*p* = 0.032). Hypertension is one of the risk factors for sarcopenia in the elderly [37]. A previous study has suggested that hypertension alters the capillarization of the muscle during aging, thereby affecting muscle function [38]. Therefore, the higher incidence of hypertension in the Sarcopenia group can be attributed to the natural course of the occurrence of sarcopenia.

The incidence of leukocytosis and antibiotic use was higher and the duration of post-ESD hospitalization was longer in the Sarcopenia group. After matching, the Sarcopenia group had a 1.23-fold higher incidence of leukocytosis (*p* < 0.049) (Figure 3). These could be the results of the pneumonia. The incidence of fever was not statically significant in the Sarcopenia group. Since fever is a non-specific symptom, the other causes of fever might have affected the result.

This study has some limitations. This was a retrospective study; therefore, it was susceptible to bias and other confounding factors. The baseline differences between the Sarcopenia and No sarcopenia groups can confound the analyses of the incidence and risk factors for pneumonia after ESD. PSM was performed to adjust the bias and other confounding factors. Although the rates of hypertension were higher in the Sarcopenia group even after matching, it can be attributed to the natural course of the development of sarcopenia during aging as described above. Another limitation is that this study only used the muscle mass from a cross-sectional area at the L3 level from CT images as the definition of sarcopenia. Sarcopenia can be diagnosed in several ways by measuring muscle mass and function, and CT images have limitations in diagnosing sarcopenia. This study is valuable in that using CT images alone for the diagnosis of sarcopenia is clinically more effective in the case of ESD as CT images are usually necessary for the evaluation of patients scheduled to undergo ESD. However, further confirmation is required to identify the exact mechanism and effect of sarcopenia on the occurrence of pneumonia after ESD.

## 5. Conclusions

In conclusion, patients with sarcopenia had an increased risk of developing pneumonia after ESD, even after adjusting for other factors. A higher risk of developing pneumonia results in a higher incidence of leukocytosis and a longer duration of post-ESD hospitalization in the Sarcopenia group. The combination of sarcopenia and age ≥73 years could be an effective predictive factor for screening groups at a high risk of developing pneumonia after ESD. Thus, the execution of strategies to reduce aspiration pneumonia may be crucial in patients aged >73 with sarcopenia, such as reduced duration of procedure and sedation, maintaining the appropriate depth of sedation, and prevention of desaturation.

## Figures and Tables

**Table 1:** Patient characteristics.

Variable	No Pneumonia (*n* = 3218)	Pneumonia (*n* = 71)	*p*-Value
Age, years	72 ± 5	74 ± 5	0.010 *
Male sex	2301 (72)	62 (87)	0.003 *
ASA physical status			0.316
I	668 (21)	13 (18)	
II	1801 (56)	36 (51)	
III	749 (23)	22 (31)	
Current smoker	1094 (34)	27 (38)	0.478
Alcohol history	1558 (48)	41 (58)	0.120
Comorbidities			
Hypertension	1796 (56)	40 (56)	0.930
Diabetes mellitus	769 (24)	17 (24)	0.993
Hepatitis	95 (3)	1 (1)	0.723
Asthma	80 (2.5)	1 (1.4)	>0.999
Bronchiectasis	7 (0.2)	0 (0)	>0.999
COPD	24 (0.8)	1 (1.4)	0.422
Old tuberculosis	188 (5.8)	4 (5.6)	>0.999
Pneumoconiosis	1 (0.03)	0 (0)	>0.999
Body mass index, kg/m^2^	24.1 ± 3.0	24.7 ± 3.3	0.107
Skeletal muscle index, cm^2^/m^2^	48.9 ± 7.9	49.4 ± 7.9	0.582
Procedure time, min	43 ± 30	51 ± 44	0.155
Recovery time, min	25 ± 10	29 ± 18	0.084
Tumor size, mm	15.8 ± 9.7	17.9 ± 10.3	0.088
Gross type of lesion			0.812
Elevated	2432 (76)	52 (73)	
Flat	415 (13)	11 (15)	
Depressed	371 (12)	8 (11)	
Location of lesion			0.075
Upper	387 (12)	3 (4)	
Middle	738 (23)	14 (20)	
Lower	2093 (65)	54 (76)	
En block resection	3113 (97)	68 (96)	0.506
Submucosal fibrosis	817 (25)	17 (24)	0.782
Sedation by anesthesiologist	3014 (94)	71 (100)	0.021 *
Fever	444 (14)	60 (85)	<0.001 *
Leukocytosis	1135 (35)	65 (92)	<0.001 *
Antibiotics use	821 (26)	71 (100)	<0.001 *
Perforation	44 (1)	3 (4)	0.080
Pneumonia			
Rt	0 (0)	4 (6)	<0.001 *
Lt	0 (0)	60 (85)	<0.001 *
Both	0 (0)	7 (10)	<0.001 *
Sarcopenia	1527 (48)	44 (62)	0.015 *
Post-ESD hospital stay, days	2.4 ± 1.0	7.8 ± 3.1	<0.001 *

Values are presented as the mean ± standard deviation or number of patients (proportion). * *p* < 0.05; ASA, American Society of Anesthesiologists; ESD, endoscopic submucosal dissection; Chronic obstructive pulmonary disease COPD.

**Table 2:** Demographic characteristics before and after matching in patients of with and without sarcopenia.

Variables	Before Matching			After Matching		
	No Sarcopenia (*n* = 1718)	Sarcopenia (*n* = 1571)	*p*-Value	No Sarcopenia (*n* = 785)	Sarcopenia (*n* = 785)	*p*-Value
Age	71 ± 5	73 ± 5	<0.001 *	72 ± 5	72 ± 5	0.367
Male sex	1008 (59)	1355 (86)	<0.001 *	604 (77)	602 (77)	0.891
ASA physical status			0.485			0.704
I	364 (21)	317 (20)		166 (21)	158 (20)	
II	965 (56)	872 (56)		450 (57)	439 (56)	
III	389 (23)	382 (24)		169 (22)	188 (24)	
Current smoker	469 (27)	652 (42)	<0.001 *	281 (36)	286 (36)	0.790
Alcohol history	689 (40)	910 (58)	<0.001 *	397 (51)	409 (52)	0.537
Comorbidities						
Hypertension	989 (58)	847 (54)	0.035 *	418 (53)	460 (59)	0.032 *
Diabetes mellitus	423 (25)	363 (23)	0.309	192 (24)	186 (24)	0.722
Hepatitis	55 (3)	41 (3)	0.314	24 (3)	24 (3)	>0.999
Asthma	44 (3)	37 (2)	0.704	14 (2)	13 (2)	0.842
Bronchiectasis	5 (0.3)	2 (0.1)	0.456	2 (0.3)	1 (0.1)	0.564
COPD	8 (0.5)	17 (1.1)	0.042 *	4 (0.5)	7 (0.9)	0.366
Old tuberculosis	69 (4.0)	123 (7.8)	<0.001 *	36 (4.6)	38 (4.8)	0.806
Pneumoconiosis	1 (0.1)	0 (0)	>0.999	1 (0.1)	0 (0)	0.317
BMI, kg/m^2^	25.6 ± 2.9	22.9 ± 2.6	<0.001 *	24.1 ± 2.4	24.1 ± 2.4	0.723
SMI, cm^2^/m^2^	52.7 ± 7.8	44.8 ± 5.7	<0.001 *	54.0 ± 7.0	44.7 ± 6.1	<0.001 *
Procedure time	42 ± 31	45 ± 30	0.013 *	44 ± 32	44 ± 30	0.628
Recovery time	24.9 ± 10.6	24.9 ± 9.7	0.982	24.4 ± 9.6	25.0 ± 9.4	0.246
Tumor size, mm	15.8 ± 10.0	15.8 ± 9.4	0.874	15.9 ± 9.5	15.2 ± 8.8	0.121
Gross type of lesion			0.029 *			0.996
Elevated	1321 (77)	1163 (74)		595 (76)	596 (76)	
Flat	197 (11)	229 (15)		99 (13)	98 (12)	
Depressed	200 (12)	179 (11)		91 (12)	91 (12)	
Location of lesion			0.081			0.405
Upper	194 (11)	196 (12)		101 (13)	88 (11)	
Middle	372 (22)	380 (24)		187 (24)	177 (23)	
Lower	1152 (67)	995 (63)		497 (63)	520 (66)	
En block resection	1666 (97)	1515 (96)	0.387	760 (97)	765 (97)	0.446
Submucosal fibrosis	404 (24)	430 (27)	0.011 *	204 (26)	201 (26)	0.863
Sedation by anesthesiologist	1613 (94)	1472 (94)	0.822	738 (94)	737 (94)	0.916
Fever	246 (14)	258 (16)	0.094	96 (12)	120 (15)	0.078
Leukocytosis	599 (35)	601 (38)	0.044 *	265 (34)	303 (39)	0.048 *
Antibiotics use	446 (26)	446 (28)	0.118	179 (23)	217 (28)	0.026 *
Perforation	22 (1)	25 (2)	0.453	13 (2)	14 (2)	0.847
Pneumonia	27 (2)	44 (3)	0.015 *	10 (1)	23 (3)	0.024 *
Rt	1 (0)	3 (0)	0.354	1 (0)	1 (0)	>0.999
Lt	24 (1)	36 (2)	0.556	7 (1)	19 (2)	0.019 *
Both	2 (0)	5 (0)	0.270	2 (0)	3 (0)	0.655
Post-ESD hospital stay	2 ± 1	3 ± 2	0.011 *	2 ± 1	3 ± 1	0.012 *

Values are presented as the mean ± standard deviation or number of patients (proportion). * *p* < 0.05; ASA, American Society of Anesthesiologists; ESD, endoscopic submucosal dissection; BMI, body mass index; SMI, skeletal muscle index; COPD, chronic obstructive pulmonary disease.

**Table 3:** Incidence and odds ratio of pneumonia based on the presence of sarcopenia and age over 73.

	Total Patients, *n*	Pneumonia Incidence, *n* (%)	Odds Ratio	95% CI	*p*-Value
No sarcopenia	785	10 (1.3%)	1 (ref)		
Sarcopenia and age < 73	453	7 (1.6%)	1.22	[0.46–3.22]	0.693
Sarcopenia and age ≥73	332	16 (4.8%)	3.92	[1.79–8.74]	0.001 *

CI, confidence interval. * *p* < 0.05.

## Data Availability

The original contributions presented in this study are included in this article. Further inquiries can be directed to the corresponding authors.

## References

[1] Oda I., Gotoda T., Hamanaka H., Eguchi T., Saito Y., Matsuda T., Bhandari P., Emura F., Saito D., Ono H. (2005). Endoscopic submucosal dissection for early gastric cancer: Technical feasibility, operation time and complications from a large consecutive series. Dig. Endosc..

[2] Isomoto H., Shikuwa S., Yamaguchi N., Fukuda E., Ikeda K., Nishiyama H., Ohnita K., Mizuta Y., Shiozawa J., Kohno S. (2009). Endoscopic submucosal dissection for early gastric cancer: A large-scale feasibility study. Gut.

[3] Park C.H., Lee H., Kim D.W., Chung H., Park J.C., Shin S.K., Hyung W.J., Lee S.K., Lee Y.C., Noh S.H. (2014). Clinical safety of endoscopic submucosal dissection compared with surgery in elderly patients with early gastric cancer: A propensity-matched analysis. Gastrointest. Endosc..

[4] Lin J.P., Zhang Y.P., Xue M., Chen S.J., Si J.M. (2015). Endoscopic submucosal dissection for early gastric cancer in elderly patients: A meta-analysis. World J. Surg. Oncol..

[5] Yamamoto H. (2012). Endoscopic submucosal dissection—Current success and future directions. Nat. Rev. Gastroenterol. Hepatol..

[6] Probst A., Golger D., Arnholdt H., Messmann H. (2009). Endoscopic submucosal dissection of early cancers, flat adenomas, and submucosal tumors in the gastrointestinal tract. Clin. Gastroenterol. Hepatol..

[7] Wang J., Zhang X.H., Ge J., Yang C.M., Liu J.Y., Zhao S.L. (2014). Endoscopic submucosal dissection vs endoscopic mucosal resection for colorectal tumors: A meta-analysis. World J. Gastroenterol..

[8] Ahn J.Y., Jung H.Y., Choi K.D., Choi J.Y., Kim M.Y., Lee J.H., Choi K.S., Kim D.H., Song H.J., Lee G.H. (2011). Endoscopic and oncologic outcomes after endoscopic resection for early gastric cancer: 1370 cases of absolute and extended indications. Gastrointest. Endosc..

[9] Saito I., Tsuji Y., Sakaguchi Y., Niimi K., Ono S., Kodashima S., Yamamichi N., Fujishiro M., Koike K. (2014). Complications related to gastric endoscopic submucosal dissection and their managements. Clin. Endosc..

[10] Oda I., Suzuki H., Nonaka S., Yoshinaga S. (2013). Complications of gastric endoscopic submucosal dissection. Dig. Endosc..

[11] Park C.H., Kim H., Kang Y.A., Cho I.R., Kim B., Heo S.J., Shin S., Lee H., Park J.C., Shin S.K. (2013). Risk factors and prognosis of pulmonary complications after endoscopic submucosal dissection for gastric neoplasia. Dig. Dis. Sci..

[12] Togo M., Akazawa Y., Akashi T., Yamashita R., Yoshitomi I., Ohba K., Hashimoto S., Iwashita H., Kurogi T., Osada Y. (2020). Comprehensive prospective analysis of the factors contributing to aspiration pneumonia following endoscopic submucosal dissection in patients with early gastric neoplasms. Acta Med. Okayama.

[13] Isomoto H., Ohnita K., Yamaguchi N., Fukuda E., Ikeda K., Nishiyama H., Akiyama M., Ozawa E., Nakao K., Kohno S. (2010). Clinical outcomes of endoscopic submucosal dissection in elderly patients with early gastric cancer. Eur. J. Gastroenterol. Hepatol..

[14] Okazaki T., Ebihara S., Mori T., Izumi S., Ebihara T. (2020). Association between sarcopenia and pneumonia in older people. Geriatr. Gerontol. Int..

[15] Niederman M.S., Cilloniz C. (2022). Aspiration pneumonia. Rev. Esp. Quimioter..

[16] Mori T., Izumi S., Suzukamo Y., Okazaki T., Iketani S. (2019). Ultrasonography to detect age-related changes in swallowing muscles. Eur. Geriatr. Med..

[17] Elliott J.E., Greising S.M., Mantilla C.B., Sieck G.C. (2016). Functional impact of sarcopenia in respiratory muscles. Respir. Physiol. Neurobiol..

[18] Cruz-Jentoft A.J., Bahat G., Bauer J., Boirie Y., Bruyère O., Cederholm T., Cooper C., Landi F., Rolland Y., Sayer A.A. (2019). Sarcopenia: Revised european consensus on definition and diagnosis. Age Ageing.

[19] Walston J.D. (2012). Sarcopenia in older adults. Curr. Opin. Rheumatol..

[20] Sheetz T., Lee C.T. (2018). Frailty and geriatric assessment in urologic oncology. Curr. Opin. Urol..

[21] Arao M., Mizutani T., Ozawa N., Hanai T., Takada J., Kubota M., Imai K., Ibuka T., Shiraki M., Araki H. (2021). Skeletal muscle depletion: A risk factor for pneumonia following gastric endoscopic submucosal dissection in elderly patients. Dig. Dis..

[22] Kim G.H., Choi K.D., Ko Y., Park T., Kim K.W., Park S.Y., Na H.K., Ahn J.Y., Lee J.H., Jung K.W. (2021). Impact of comorbidities, sarcopenia, and nutritional status on the long-term outcomes after endoscopic submucosal dissection for early gastric cancer in elderly patients aged ≥80 years. Cancers.

[23] Ito N., Funasaka K., Miyahara R., Furukawa K., Yamamura T., Ishikawa T., Ohno E., Nakamura M., Kawashima H., Hirooka Y. (2022). Relationship between psoas muscle index and long-term survival in older patients aged ≥80 years after endoscopic submucosal dissection for gastric cancer. Int. J. Clin. Oncol..

[24] Bahat G., Turkmen B.O., Aliyev S., Catikkas N.M., Bakir B., Karan M.A. (2021). Cut-off values of skeletal muscle index and psoas muscle index at l3 vertebra level by computerized tomography to assess low muscle mass. Clin. Nutr..

[25] Aubrey J., Esfandiari N., Baracos V.E., Buteau F.A., Frenette J., Putman C.T., Mazurak V.C. (2014). Measurement of skeletal muscle radiation attenuation and basis of its biological variation. Acta Physiol..

[26] Prado C.M., Lieffers J.R., McCargar L.J., Reiman T., Sawyer M.B., Martin L., Baracos V.E. (2008). Prevalence and clinical implications of sarcopenic obesity in patients with solid tumours of the respiratory and gastrointestinal tracts: A population-based study. Lancet Oncol..

[27] Martin L., Birdsell L., Macdonald N., Reiman T., Clandinin M.T., McCargar L.J., Murphy R., Ghosh S., Sawyer M.B., Baracos V.E. (2013). Cancer cachexia in the age of obesity: Skeletal muscle depletion is a powerful prognostic factor, independent of body mass index. J. Clin. Oncol..

[28] Iritani S., Imai K., Takai K., Hanai T., Ideta T., Miyazaki T., Suetsugu A., Shiraki M., Shimizu M., Moriwaki H. (2015). Skeletal muscle depletion is an independent prognostic factor for hepatocellular carcinoma. J. Gastroenterol..

[29] Miyata H., Sugimura K., Motoori M., Fujiwara Y., Omori T., Yanagimoto Y., Ohue M., Yasui M., Miyoshi N., Tomokuni A. (2017). Clinical assessment of sarcopenia and changes in body composition during neoadjuvant chemotherapy for esophageal cancer. Anticancer Res..

[30] Mandell L.A., Niederman M.S. (2019). Aspiration pneumonia. N. Engl. J. Med..

[31] Dennison E.M., Sayer A.A., Cooper C. (2017). Epidemiology of sarcopenia and insight into possible therapeutic targets. Nat. Rev. Rheumatol..

[32] Tang D., Yuan F., Ma X., Qu H., Li Y., Zhang W., Ma H., Liu H., Yang Y., Xu L. (2021). Incidence rates, risk factors, and outcomes of aspiration pneumonia after gastric endoscopic submucosal dissection: A systematic review and meta-analysis. J. Gastroenterol. Hepatol..

[33] Kim J.W., Kim R., Choi H., Lee S.J., Bae G.U. (2021). Understanding of sarcopenia: From definition to therapeutic strategies. Arch. Pharm. Res..

[34] Chen M., Wang Y., Deng S., Lian Z., Yu K. (2022). Skeletal muscle oxidative stress and inflammation in aging: Focus on antioxidant and anti-inflammatory therapy. Front. Cell Dev. Biol..

[35] Bauer J., Biolo G., Cederholm T., Cesari M., Cruz-Jentoft A.J., Morley J.E., Phillips S., Sieber C., Stehle P., Teta D. (2013). Evidence-based recommendations for optimal dietary protein intake in older people: A position paper from the prot-age study group. J. Am. Med. Dir. Assoc..

[36] Kaplan S.A., Lee J.Y., O’Neill E.A., Meehan A.G., Kusek J.W. (2013). Prevalence of low testosterone and its relationship to body mass index in older men with lower urinary tract symptoms associated with benign prostatic hyperplasia. Aging Male.

[37] He N., Zhang Y., Zhang L., Zhang S., Ye H. (2021). Relationship between sarcopenia and cardiovascular diseases in the elderly: An overview. Front. Cardiovasc. Med..

[38] Gueugneau M., Coudy-Gandilhon C., Meunier B., Combaret L., Taillandier D., Polge C., Attaix D., Roche F., Feasson L., Barthelemy J.C. (2016). Lower skeletal muscle capillarization in hypertensive elderly men. Exp. Gerontol..

